# Soft skills and potential covariates in first year health science students in Latin America: a cross-sectional study

**DOI:** 10.3389/fpsyg.2026.1845073

**Published:** 2026-06-17

**Authors:** Víctor P. Díaz-Narváez, Fernando Reyes-Reyes, Alejandro Reyes-Reyes, J. E. Rod, José Gamarra-Moncayo, Oscar Emilio Hernández Bustos, Ayleen Indira Lubo Gelvez

**Affiliations:** 1 Departamento de Investigación, Facultad de Odontología, Universidad Andres Bello, Santiago, Chile; 2 Facultad de Psicología, Instituto de Bienestar Socioemocional, Universidad del Desarrollo, Concepción, Chile; 3 Facultad de Ciencias Sociales y Comunicación, Universidad Santo Tomás, Concepción, Chile; 4 División de Ciencias de la Salud, Departamento de Salud Pública, Universidad del Norte, Barranquilla, Colombia; 5 Facultad de Medicina, Universidad Católica Santo Toribio de Mogrovejo, Chiclayo, Peru; 6 Institución Universitaria de Barranquilla, Barranquilla, Colombia; 7 Facultad de Medicina, Universidad del Norte, Barranquilla, Colombia

**Keywords:** global south, humanization, non-cognitive traits, socio-emotional competency, undergraduate

## Abstract

**Background:**

Latin American health education institutions traditionally prioritize technical skills over “soft” psychosocial competencies. However, empathy and resilience are vital for quality clinical care. This study aimed to explore the psychometric properties, gender differences, and correlations between soft skills (empathy, resilience) and psychosocial covariates (affect, family functioning, satisfaction with life) in first-year students.

**Methods:**

A quantitative, cross-sectional study was conducted with a convenience sample of 896 first-year students (59% female, 41% male) from Dentistry, Kinesiology, and Nursing programs at three universities in Chile. Participants completed five validated instruments: JSE-HPS (Empathy), EEA (Resilience), PANAS (positive and negative affect), FACES-20 (Family Functioning), and SWLS (Satisfaction with Life). Data were analyzed using Confirmatory Factor Analysis (CFA) and Pearson correlations.

**Results:**

CFA confirmed adequate structural validity and reliability for most scales, though gender invariance was only strictly met for affect and family functioning. Significant gender differences were observed: men scored higher in compassionate care, positive affect, and engineering/adaptive resilience, while women scored higher in perspective taking and negative affect. Resilience showed moderate positive correlations with positive affect (*r* = 0.401) and satisfaction with life (*r* = 0.368). Overall, students scored in the mean range, suggesting a developmental deficit in these competencies upon university entry.

**Conclusion:**

The study provides a psychometric foundation for assessing soft skills in Latin American health students. The results highlight the need for early risk gender and sub dimensions adjusted profiling to identify students more susceptible of soft skill decline during training. Furthermore, further research is needed to develop training interventions to improve soft skills during academic years.

## 1 Introduction

Latin American universities select undergraduate health science students through entrance examinations that measure technical knowledge, the so-called “hard” skills (Calles Santoyo et al., 2025; Martínez-González et al., 2021). However, assessments of “soft” skills at the onset of their studies are infrequent (Gamarra Aparicio, 2025). Healthcare inherently requires direct engagement with patients and their families, therefore cultivating these competencies is vital for enhancing quality clinical care and patient satisfaction (Sancho-Cantus et al., 2023). Currently, there is a prominent trend toward the integrated inclusion (at least theoretically) of both “cognitive” and “emotional” dimensions within teaching-learning processes. These aspects constitute inseparable elements of a system that actively interact in the processes of knowledge appropriation and internalization (Ippolito and Kingsbury, 2024; Reisenzein, 2019). The most widely accepted theoretical foundations of this perspective are rooted in the works of Piaget (Bjorklund, 2018) and Vygotsky (Lane and Smith, 2021) with empirical evidence further strengthening these theories (Lamri and Lubart, 2023; Lane, 2008; Ratner et al., 2002). Therefore, claiming that one type of skill is superior to the other creates a false dichotomy regarding the necessity of both hard and soft skill development in the comprehensive training of health sciences students (Lamri and Lubart, 2023). The overemphasis on hard skills within teaching-learning processes represents a reductionist view of student formation in the health sciences (Díaz-Narváez and Calzadilla-Núñez, 2001).

The need to assess soft skills at the beginning of health science training can also be framed within contemporary models of health professions education. Competency-based education proposes that professional training should not be restricted to the acquisition of disciplinary knowledge, but should focus on the progressive development of observable, contextualized, and socially accountable competencies required for effective professional performance (Frank et al., 2010; Gruppen et al., 2012). From this perspective, professional competence involves the habitual and judicious integration of knowledge, technical skills, communication, emotions, values, reflection, and clinical reasoning for the benefit of individuals and communities (Epstein and Hundert, 2002). International competency frameworks, such as CanMEDS, similarly conceptualize health professionals not only as technical experts, but also as communicators, collaborators, health advocates, and professionals, roles that require socio-emotional and interpersonal capacities closely related to empathy and resilience (Frank et al., 2015). In addition, cultural safety and critical curriculum perspectives emphasize that health professional education should prepare students to recognize power asymmetries, social inequities, and the hidden curriculum that may shape professional identity, patient interaction, and emotional responses during training (Curtis et al., 2019; Hafferty, 1998). Therefore, evaluating empathy, resilience, affective functioning, family functioning, and satisfaction with life upon university entry provides a theoretically grounded baseline for understanding how students begin their professional formation and how curricula may later strengthen or erode these competencies.

In this sense, there are different classifications of what is conceptually represented in the label “soft skills.” However, they are usually categorized as: personal (behavioral, psychological), social and methodological skills (Continisio et al., 2021; Naamati-Schneider and Alt, 2025). These attributes facilitate human interaction, professional performance, and personal management in any field. However, the construction of the theory of skills is not yet fully developed and, therefore, there is no clarity as to which constructs or attributes influence the positive or negative development of these skills. The quality of the comprehensive training of undergraduate and postgraduate health sciences students increases when training includes the teaching and learning of soft skills (Lamiani et al., 2021; Ramollari and Kontodimopoulos, 2025; Zimonjić et al., 2025).

Two relevant soft skills emphasized in international competency frameworks for health professionals are empathy and resilience (Díaz-Narváez et al., 2025a,b,c; Di Giacomo et al., 2025; Jardim et al., 2022). It has been suggested that factors such as positive and negative affect, satisfaction with life, and family functioning may be related to the development of resilience (Haider et al., 2022; Liu et al., 2024; Wang et al., 2019). However, there is a lack of literature regarding how psychological factors might influence empathy. Additionally, interest in the potential determinants of empathy among health science students has primarily focused on understanding the factors that drive empathy decline during undergraduate training (Barker et al., 2022; Chhabra et al., 2022; Gilligan et al., 2021; Lumish et al., 2022). To the best of our knowledge, there are no previous studies specifically looking at psychosocial factors influencing resilience and empathy in first year health science students.

Most studies concerning the status of soft skills, or specific constructs within this domain, have been conducted while students are in their preclinical and clinical years (García-Lancaster et al., 2022; Leo Ramírez et al., 2019; Perrot Tabilo et al., 2024). Generally, and specifically within Latin America, there is a scarcity of research analyzing in detail the situation of health science students as they first enter university. Studies of this nature have immediate practical application, as they provide robust data on the behavior of constructs that significantly explain the state of students’ soft skills upon university entry. Consequently, assessing these constructs may contribute to the early identification of psychosocial profiles potentially associated with later vulnerability to burnout, academic disengagement, or attrition. From a pedagogical standpoint, this enables health faculties to implement proactive curricular interventions and support strategies that strengthen socio-emotional competencies from the outset, ultimately improving educational outcomes and fostering more resilient, humanized professional training.

## 2 Objectives

The aims of the present study were to (i) explore the psychometric properties, construct validity of the psychosocial factors of affect, family function, satisfaction with life and the soft skills of resilience and empathy in health science students at the beginning of their first year of study, (ii) explore sex differences among the psychological sub-dimensions of each scale and (iii) explore the correlation between the psychosocial factors and soft skills.

## 3 Methods

### 3.1 Design, procedure and study participants

A quantitative, cross-sectional, and exploratory research approach was used. Student participation was voluntary. This approach is based on the principle that psychological questionnaires must be administered under the concept of voluntariness to ensure honesty and validity of responses, protect participant privacy, and guarantee emotional well-being. For these reasons, random sampling was not possible. Students signed an informed consent form before completing the instruments, thereby guaranteeing the confidentiality of their information. Furthermore, the same general pedagogical approach was applied to all participants, with variations occurring only in the specific methods and didactics of each major. Data collection took place in June and July 2024.

The instruments were administered in a group setting, in a pen-and-paper format, during regular class hours but within an academic environment. The instruments were collected by professors from the Faculties of Health Sciences of the participating universities; these professors were not active or passive participants in this research but received the necessary training to provide the instruments, answer questions strictly regarding how to mark responses, and ensure the collection of the materials. Professors were strictly prohibited from providing any personal interpretation regarding the questions or their content.

The total population of first-year students across the three majors and the three examined universities was 1,563 (*N*). We collected a convenience sample that consisted of 896 (57.01% of the total population) Chilean first-year students from three health science majors, with ages ranging from 17 to 38 years (*M* = 19.44, SD = 1.81), of whom 59% were women and 41% were men. The distribution by major was as follows: Dentistry (51.4%), Kinesiology (34.8%), and Nursing (13.8%), belonging to Andrés Bello University (Santiago, Viña del Mar, and Concepción campuses), Universidad del Desarrollo, and Universidad Santo Tomás (Concepción campus).

### 3.2 Instruments

Before administration, all scales were reviewed by experts. They evaluated the translation and back-translation processes carried out by the Spanish and English faculty, respectively. Once the translation and back-translation results were approved, the experts examined the content of the instrument in Spanish and conducted a pilot test with a randomly selected group, including first-year students, to ensure the clarity and comprehensibility of the questions in both versions.

#### 3.2.1 Jefferson Scale of Empathy Health Professions Students version (JSE-HPS)

Consisting of 20 items, it measures empathy levels toward patients in health sciences students of any specialty. Items are rated on a 7-point Likert scale, ranging from 1 (strongly disagree) to 7 (strongly agree). The instrument measures three dimensions: Compassionate Care (CC; items 1, 7, 8, 11, 12, 14, 18, 19), Perspective Adoption (PA; items 2, 4, 5, 9, 10, 13, 15, 16, 17, 20), and ‘Walking in the Patient’s Shoes’ (WIPS; items 3 and 6). The scale has demonstrated adequate internal consistency (alpha = 0.78–0.92) and appropriate correlations with other psychological variables (Hojat et al., 2001). The JSE was used in this study with permission from Thomas Jefferson University.

#### 3.2.2 Resilience Trait Scale (EEA)

It evaluates three dimensions of resilience: engineering, ecological and adaptive. It has a 12-item Likert-type format, with five response levels per item, from “Strongly disagree” (1) to “Strongly agree.” (5). It has demonstrated adequate internal and test–retest reliability, a cross-culturally stable factor structure, convergent and construct validity in terms of associations with personality, and a positive contribution to clinical and non-clinical psychological health states. The resilience scale is composed of three distinct sub-dimensions: (i) Engineering (ENG), which focuses on the ability to recover from stressful events with ease; (ii) Ecological (ECO), which reflects the capacity to remain strong and (iii) Adaptation (ADA), spanning which measures the extent to which uncertain situations pique an individual’s interest (Maltby et al., 2016; Maltby et al., 2015).

#### 3.2.3 Positive and negative affect (PANAS)

It consists of two mood scales: one measuring positive affect and another measuring negative affect. It comprises 20 items using a 5-point scale ranging from ‘Very slightly or not at all’ (1) to ‘Extremely’ (5). Used as a psychometric scale, the PANAS can demonstrate the relationships between positive and negative affect. Ten descriptors are used for each scale: Positive Affect (PA) and Negative Affect (NA). Cronbach’s Alpha coefficients for positive and negative affect typically range between 0.86–0.90 and 0.84–0.87, respectively. Test–Retest: It has shown moderate to good correlations over several weeks, confirming its stability as a measure of affective states. The two-factor model (positive affect and negative affect) has been confirmed with good model fit and correlates well with other measures of anxiety and depression (Hovmand et al., 2023). Cross-cultural validity has also been established (Chile, Korea, India) (Kumar et al., 2025; Lim et al., 2010; Sortheix and Weber, 2023; Vera-Villarroel et al., 2019).

#### 3.2.4 Family Functioning Scale (FACES-20 Esp)

The abbreviated 20-item Spanish Family Adaptability and Cohesion Evaluation Scales were used to evaluate family functioning. Each item describes a family situation using a Likert scale from 0 to 4 (never to almost always). This version has been validated in Spain (Martínez-Pampliega et al., 2011), Chile (Zicavo et al., 2012), and Ecuador (Dávila Pontón et al., 2019), as well as in Latin American health sciences students (Vilca et al., 2024).

#### 3.2.5 Satisfaction with Life Scale (SWLS)

This is a brief 5-item questionnaire designed to measure a person’s global cognitive assessment of their own life. It uses a Likert-type scale typically ranging from 1 (Strongly disagree) to 7 (Strongly agree). It evaluates subjective well-being, possesses the capacity to predict future behaviors, and is well-correlated with mental health (Dávila Pontón et al., 2019). The scale’s alpha coefficient fluctuates between 0.79 and 0.89, indicating high internal consistency (Rogowska et al., 2021; Valenti and Faraci, 2024). The scale has also been found to have good test–retest correlations (0.84 and 0.80 over a one-month interval) (Baattaiah et al., 2023; Laranjeira, 2009). Similar results regarding construct validity and invariance have also been found in Chile (Bagherzadeh et al., 2018; Schnettler Morales et al., 2014).

### 3.3 Statistical analysis

The final sample (*n* = 896) was subjected to multivariate outlier analysis (*p* < 0.001) using Mahalanobis distances (Hair et al., 2018). No outliers were found. Univariate descriptive statistics were calculated for the five instruments (empathy, positive and negative affect, family functioning, resilience, and satisfaction with life). Subsequently, Confirmatory Factor Analyses (CFA) were performed using the Robust Maximum Likelihood (MLR) estimator for empathy and satisfaction with life, as they feature seven response options, allowing them to be treated as continuous variables (Rhemtulla et al., 2012). In contrast, the Weighted Least Squares Mean and Variance Adjusted (WLSMV) estimator was chosen for positive and negative affect, family functioning, and resilience, as it is recommended for the analysis of ordinal variables based on polychoric correlation matrices (Kline, 2023) and these instruments consist of five response options. Furthermore, robust methods are crucial when deviations from multivariate normality occur.

The cutoff points considered for the fit indices were: Comparative Fit Index (CFI > 0.90), Tucker-Lewis Index (TLI > 0.90), Root Mean Square Error of Approximation (RMSEA < 0.08), and Standardized Root Mean Square Residual (SRMR < 0.05) (Whittaker and Schumacker, 2022). For internal consistency, the Omega coefficient was calculated (Viladrich et al., 2017). Measurement invariance was corroborated by sex and academic major, considering the cutoff points for Delta Comparative Fit Index (Delta CFI ≤ −0.01) and Delta Root Mean Square Error of Approximation (Delta RMSEA ≥ 0.015) (Chen, 2007). Additionally, differences by sex were verified using Student’s *t*-test for independent samples—given its robustness to deviations from normality in large samples (Fagerland, 2012) and the effect size was estimated using Cohen’s *d* (Domínguez-Lara, 2018). Moreover, cutoff points were established based on percentiles (Muñiz, 2018), considering a classification of five categories (very high, high, medium, low, and very low). Analyses were executed using JASP (0.19.1) software and R within the RStudio interface, utilizing the packages *lavaan* (0.6–20), psych (2.5.6), and *semTools* (0.5–7).

## 4 Results

### 4.1 Psychometric properties and structural validity

The univariate analysis of the scales found that at least one item exceeds the maximum accepted values for skewness and kurtosis (±2) (Bandalos and Finney, 2019). Therefore, univariate normality is affected (see Table 1). Conversely, this is not observed in the instruments for resilience and satisfaction with life. However, the MLR and WLSMV estimators correct for this potential bias in the CFA.

**Table 1:** Univariate descriptive statistics for all scale items.

Items	*M*	*σ*	g1	g2	Items	*M*	*σ*	g1	g2
Empathy									
1	3.14	2.12	0.54	−1.11	11	2.75	1.73	0.92	−0.07
2	6.55	0.94	−3.15	12.31	12	2.23	1.59	1.41	1.10
3	3.72	1.49	0.05	−0.64	13	6.08	1.19	−1.52	2.30
4	6.44	0.96	−2.21	5.75	14	1.90	1.34	1.88	3.37
5	5.45	1.54	−0.91	0.22	15	5.34	1.62	−0.83	0.04
6	4.26	1.61	−0.17	−0.77	16	6.12	1.15	−1.49	2.27
7	1.96	1.54	1.83	2.60	17	5.71	1.34	−0.98	0.53
8	2.52	1.75	1.08	0.12	18	4.63	1.61	−0.20	−0.63
9	6.22	1.10	−1.65	2.90	19	3.07	1.75	0.47	−0.66
10	6.12	1.15	−1.51	2.15	20	6.41	1.01	−2.11	5.07
PANAS									
1	3.25	1.14	−0.34	−0.52	11	2.33	1.24	0.59	−0.70
2	2.39	1.20	0.44	−0.88	12	3.07	1.13	−0.07	−0.64
3	3.24	1.17	−0.27	−0.69	13	1.76	1.05	1.27	0.70
4	1.99	1.12	0.92	−0.09	14	3.09	1.21	−0.15	−0.82
5	3.66	1.14	−0.53	−0.48	15	2.68	1.34	0.21	−1.17
6	1.65	1.02	1.55	1.54	16	3.49	1.15	−0.47	−0.53
7	1.97	1.18	1.00	−0.05	17	3.34	1.06	−0.31	−0.40
8	1.51	0.93	1.95	3.27	18	2.05	1.15	0.89	−0.12
9	3.39	1.15	−0.30	−0.66	19	3.40	1.13	−0.22	−0.68
10	3.36	1.12	−0.20	−0.70	20	1.94	1.19	1.13	0.25
Family functioning									
1	3.33	0.83	−1.19	1.10	11	2.76	1.07	−0.58	−0.39
2	2.83	1.03	−0.71	0.02	12	2.83	1.12	−0.71	−0.37
3	3.12	0.94	−1.13	1.14	13	3.56	0.80	−2.16	4.83
4	2.90	1.02	−0.73	−0.10	14	2.90	1.09	−0.75	−0.27
5	3.20	0.98	−1.15	0.72	15	2.80	1.12	−0.71	−0.23
6	2.87	1.09	−0.80	−0.01	16	3.07	1.04	−1.00	0.33
7	2.99	0.99	−0.91	0.40	17	2.76	1.10	−0.71	−0.20
8	2.76	1.10	−0.57	−0.48	18	2.51	1.22	−0.46	−0.75
9	3.45	0.83	−1.59	2.35	19	3.18	1.00	−1.12	0.58
10	3.20	1.05	−1.25	0.84	20	2.81	1.14	−0.78	−0.19
Resilience									
1	3.55	1.12	−0.53	−0.44	7	3.90	1.02	−0.85	0.32
2	3.38	1.12	−0.29	−0.65	8	3.99	0.97	−0.91	0.60
3	3.47	1.12	−0.38	−0.62	9	3.52	1.16	−0.43	−0.53
4	3.39	1.12	−0.27	−0.71	10	3.18	1.19	−0.11	−0.78
5	4.14	0.98	−1.17	1.04	11	3.31	1.20	−0.27	−0.80
6	3.83	1.03	−0.70	−0.05	12	3.49	1.22	−0.46	−0.69
Satisfaction with life									
1	4.93	1.33	−0.55	0.17	4	5.22	1.55	−0.70	−0.26
2	5.39	1.31	−0.67	0.01	5	4.65	2.01	−0.45	−1.04
3	5.43	1.47	−0.87	0.17	—	—	—	—	—

*M*, mean; *σ*, standard deviation; g1, asymmetry; g2, kurtosis.

The CFA results evaluating the structural validity of the scales are presented in Table 2.

**Table 2:** Confirmatory factor analysis for all scales.

Scale	*X*^2^ (gl)	*p*	CFI	TLI	RMSEA [IC 90%]	SRMR
Empathy	1122.17 (169)	<0.001	0.96	0.95	0.07 [0.07-0.08]	0.05
PANAS	860.86 (170)	<0.001	0.93	0.93	0.06 [0.06-0.07]	0.08
Family functioning	358.54 (167)	<0.001	0.94	0.93	0.03 [0.03-0.04]	0.04
Resilience	380.72 (51)	<0.001	0.98	0.97	0.08 [0.07–0.09]	0.05
Life satisfaction	23.48 (5)	<0.001	0.98	0.97	0.06 [0.04–0.09]	0.02

All scales obtained adequate fit indices. Furthermore, to evaluate the internal consistency of the scales Table 3 shows the McDonald’s Omega (*ω*) where the scales demonstrated good to excellent consistency except for an empathy sub dimension called “Walking in the patient shoes” (<0.70).

**Table 3:** Scales dimensions descriptives and internal consistency.

Scale	Dimension	*M* (SD)	*ω*
Empathy	Compassionate care	22.19 (8.06)	0.74
	Perspective taking	60.42 (7.12)	0.78
	Walking in the patient’s shoes	7.98 (2.64)	0.62
PANAS	Positive affect	33.29 (7.41)	0.89
	Negative affect	20.26 (7.39)	0.87
Family functioning	Cohesion	30.44 (7.33)	0.91
	Flexibility	29.36 (7.40)	0.90
Resiliency	Engineering	14.10 (3.78)	0.92
	Ecologic	17.10 (2.50)	0.79
	Adaptation	13.99 (3.66)	0.80
Satisfaction with life^a^	—	25.61 (5.95)	0.83

a Unidimensional scale.

Table 4 shows that the instruments for positive and negative affect and family functioning proved to be strictly invariant by sex. However, the same was not true for the scales of empathy, satisfaction with life, and resilience. Also, invariance by academic program could not be demonstrated for any of the instruments. Therefore, direct comparisons of observed scores between men and women, as well as across different professional majors, should be conducted with caution.

**Table 4:** Invariance of the scales by sex.

Scale^a^	Invariance level	*X*^2^ (d*f*)	CFI	ΔCFI	RMSEA	ΔRMSEA	SRMR
Empathy	Configural	612.39 (334)	0.927		0.043		0.047
	Metric	641.28 (351)	0.924	−0.003	0.043	0	0.051
	Scalar	721.55 (368)	0.908	−0.017	0.046	0.003	0.054
	Strict	794.13 (368)	0.894	−0.014	0.048	0.002	0.059
Positive and negative affect	Configural	1604.44 (340)	0.944		0.091		0.093
	Metric	1634.93 (358)	0.944	−0.001	0.089	−0.002	0.093
	Scalar	1634.93 (356)	0.944	0	0.090	0	0.093
	Strict	1634.93 (356)	0.944	0	0.090	0	0.093
Family functioning	Configural	800.974 (338)	0.994		0.055		0.052
	Metric	933.97 (356)	0.993	−0.001	0.060	0.005	0.056
	Scalar	933.97 (354)	0.993	0	0.061	0	0.056
	Strict	933.97 (354)	0.993	0	0.061	0	0.056
Satisfaction with life	Configural	29.65 (10)	0.988		0.066		0.020
	Metric	31.14 (14)	0.990	0.002	0.052	−0.014	0.023
	Scalar	80.61 (18)	0.963	−0.027	0.088	0.036	0.044
	Strict	111.73 (23)	0.947	−0.016	0.093	0.005	0.052

a Measurement invariance for the resilience instrument could not be demonstrated starting from the configural level.

Table 5 presents cut-off points divided into five categories for the analyzed instruments. Given the presence of invariant scales, it is inferred that these cut-offs points are useful for classifying values in a manner that avoids bias by sex or academic major.

**Table 5:** Proposed cutoff points for the five scales.

Percentile	CC	PA	WIPS	POS-A	NEG-A	COH	FLE	ENG	ECO	ADA	SWLS	Level
99	47	70	14	49	39	40	40	20	20	20	35	Very high
90	33	68	13	43	31	39	38	19	19	19	33	
80	28	67	12	40	27	37	36	17	18	17	31	High
70	25	65	11	37	23	35	34	16	17	16	29	
60	23	64	10	36	21	34	32	15	16	15	28	Average
50	21	62	9	34	19	32	30	14	15	14	26	
40	19	60	8	31	17	30	29	13	14	13	25	
30	17	58	7	29	15	28	27	12	13	12	23	Low
20	15	55	6	27	13	25	24	11	12	11	21	
10	13	51	4	24	11	20	19	9	11	9	17	Very low
1	9	40	2	16	10	9	9	5	10	4	10	

CC, compassionate care; PA, perspective adoption; WIPS, walking in patients’ shoes; POS-A, positive affect; NEG-A, negative affect; COH, cohesion; FLE, flexibility; ENG, engineering; ECO, ecological; ADA, adaptation; SWLS, satisfaction with life.

### 4.2 Gender differences

Table 6 shows that significant gender differences were found in all analyzed variables, except for cohesion and flexibility (family functioning), ecological (resilience), and satisfaction with life. Men obtained significantly higher scores in the dimensions of care with compassion and walking in the patient’s shoes (empathy), positive affect, and finally, engineering and adaptation (resilience). On the other hand, differences favoring women were found in perspective taking (empathy) and negative affect. Effect sizes ranged from trivial to medium, with the most prominent being in the engineering dimension (resilience).

**Table 6:** Sex differences by sub-dimensions of each scale.

Variables	Sex	*M* (DE)	*t*	*p*	*d*	Interpretation of *d* magnitude
CC (E)	Male	24.19 (8.68)	6.30	<0.001	0.428	Small
	Female	20.81 (7.29)				
PA (E)	Male	59.57 (7.31)	−3.01	0.003	−0.205	Small
	Female	61.02 (6.93)				
WIPS (E)	Male	8.31 (2.60)	3.14	0.002	0.214	Small
	Female	7.75 (2.65)				
POS-A (PANAS)	Male	34.38 (7.22)	3.67	<0.001	0.249	Small
	Female	32.54 (7.46)				
NEG-A (PANAS)	Male	19.49 (7.02)	−2.60	0.009	0.177	Very small
	Female	20.79 (7.59)				
COH (FF)	Male	29.95 (6.81)	−1.70	0.090	0.115	Very small
	Female	30.79 (7.65)				
FLE (FF)	Male	29.31 (7.01)	−0.20	0.843	0.013	Very small
	Female	29.41 (7.66)				
ENG (R)	Male	15.32 (3.43)	8.33	<0.001	0.566	Medium size
	Female	13.26 (3.78)				
ECO (R)	Male	17.25 (2.37)	1.56	0.118	0.118	Very small
	Female	16.99 (2.58)				
ADA (R)	Male	14.73 (3.42)	5.13	<0.001	0.349	Small
	Female	13.47 (3.74)				
SAT (SWLS)	Male	25.45 (5.97)	−0.66	0.507	0.045	Very small
	Female	25.72 (5.95)				

CC, compassionate care; PA, perspective adoption; WIPS, walking in the patient’s shoes; POS-A, positive affect; NEG-A, negative affect; COH, cohesion; FLE, flexibility; ENG, engineering; ECO, ecological; ADA, adaptation; SWLS, satisfaction with life. Male (*n* = 367), female (*n* = 529).

### 4.3 Correlation between psychosocial factors and soft skills

Table 7 presents the Pearson correlation coefficients between the analyzed soft skills (empathy and resilience) and their potential covariates. The results reveal that both empathy and resilience were significantly correlated with almost all hypothesized potential predictors, providing preliminary support for their role as covariates. Specifically, Resilience showed the strongest associations, correlating moderately and positively with positive affect (*r* = 0.401, *p* < 0.01), satisfaction with life (*r* = 0.368, *p* < 0.01), and flexibility (*r* = 0.314, *p* < 0.01), while showing a significant inverse relationship with negative affect (*r* = −0.229, *p* < 0.01).

**Table 7:** Correlations between potential covariates and soft skills.

Variables	Soft skills		Potential covariates of soft skills				
	1	2	3	4	5	6	7
1. Empathy	1						
2. Resilience	184^**^	1					
3. Cohesion	0.111^**^	0.270^**^	1				
4. Flexibility	0.137^**^	0.314^**^	0.835^**^	1			
5. Satisfaction with life	0.139^**^	0.368^**^	0.435^**^	0.438^**^	1		
6. Positive affects	0.201^**^	0.401^**^	0.210^**^	0.245^**^	0.416^**^	1	
7. Negative affects	0.082^*^	−0.229^**^	−0.103^**^	−0.131^**^	−0.188^**^	−0.033	1

In contrast, the correlations for empathy were generally weaker; it was positively associated with positive affect (*r* = 0.201, *p* < 0.01), satisfaction with life (*r* = 0.139, *p* < 0.01), and flexibility (*r* = 0.137, *p* < 0.01), though it also showed a weak but significant positive correlation with negative affect (*r* = 0.082, *p* < 0.05). Regarding inter-variable relationships, the two dimensions of family functioning, cohesion and flexibility exhibited a very strong positive correlation (*r* = 0.835, *p* < 0.01), suggesting high multicollinearity between these potential predictors, while Empathy and Resilience themselves showed a significant but low positive correlation (*r* = 0.184, *p* < 0.01).

## 5 Discussion

### 5.1 Psychometrics and neurobiological basis

Psychometric analysis in specific populations ensures data distribution accuracy, model compliance, and invariance, eliminating systematic biases (Díaz-Narváez et al., 2024). Our results confirm a theoretical model fit and reliability across five constructs, excluding the SIPS sub-dimension of empathy. Gender invariance was established for positive/negative affect and family functioning, validating cross-gender comparisons regarding structure, meaning, and precision (Romppel et al., 2017). In contrast there was a lack of invariance across academic majors., Furthermore, while PANAS gender invariance has been documented globally (Buz et al., 2015; Costa et al., 2020; Veronese and Pepe, 2017), Latin American studies remain scarce and often yield partial results influenced by item-specific emotional nuances (Martín-Carbonell et al., 2021; Parra-Gaete et al., 2025). Although PANAS is frequently used for criterion validity alongside emotional regulation or stress (Aragón et al., 2026; Hofer et al., 2007; Ma et al., 2025), invariance findings regarding affect expression remain inconclusive due to contextual and cohort-specific factors (Batista et al., 2020; Guse and van Zyl, 2021).

In this study, males reported slightly higher positive affect, while negative affect showed no significant gender disparity, possibly due to the shared high academic pressure among healthy students. Robust Latin American psychometric studies are required to enable empirical generalizations (Batista et al., 2020; Guse and van Zyl, 2021). Recent research (2023–2026) demonstrates that positive/negative affect, empathy, resilience, family functioning, and satisfaction with life share integrated prefrontal-limbic circuits and large-scale brain networks (Bickart et al., 2023). For instance, for positive/negative affect and regulation, the dopaminergic reward circuits mediate positive affect (Ge et al., 2025; Savalia et al., 2025; Shin et al., 2024), while amygdala hyperactivity and prefrontal dysregulation drive negative affect (Bickart et al., 2023; Feng et al., 2024; Sultana et al., 2023; Wu et al., 2024). PFC-amygdala connectivity determines effective emotional regulation (Compère et al., 2023; Fang et al., 2024; Kim and Kim, 2024; Sugiura et al., 2023; Kremer et al., 2024). In the case of empathy and resilience, the mentalization network (medial prefrontal cortex, temporoparietal junction and superior temporal sulcus) and mirror neuron system support empathy (Ma et al., 2024; McDonald et al., 2024; Konrad et al., 2024; Wang et al., 2024) while resilience is characterized by hippocampal neuroplasticity, and emotional granularity which acts as a protective substrate against adversity (Compère et al., 2023; Kim and Kim, 2024; Shin et al., 2024; Weissman et al., 2024). In the case of family satisfaction and cohesion, it is reflected in interpersonal neural synchrony within the medial prefrontal cortex and superior temporal sulcus, modulated by oxytocin (Chen et al., 2025; Kremer et al., 2024; Zhou et al., 2023). Satisfaction with life correlates with ventral striatum activation, default mode network connectivity, and prefrontal cortical thickness (Kremer et al., 2024; Li et al., 2024; Lettieri et al., 2024; Park et al., 2023; Racicot et al., 2024; Shin et al., 2024).

These constructs form an integrated system converging at the prefrontal-amygdala-striatum-hippocampus axis (Ge et al., 2025; Kim and Kim, 2024; Kremer et al., 2024; Shin et al., 2024). They do not function autonomously; rather, they rely on shared mechanisms for emotional processing and reward (Kim and Kim, 2024; Konrad et al., 2024; Li et al., 2024; Lettieri et al., 2024; Ma et al., 2024; Racicot et al., 2024; Sultana et al., 2023). The student’s capacity for resilience and empathy depends on the same PFC-limbic functional connectivity, while satisfaction with life is intertwined with dopaminergic pathways. This structure can be considered a “social brain” that synchronizes with the family environment through shared rhythmic brain activity, suggesting that early dysfunction or enhancement within these networks concurrently impacts all psychological domains (Ge et al., 2025; Kim and Kim, 2024; Li et al., 2024). This implies interventions aiming at enhancing one construct could enhance simultaneously multiple constructs (Compère et al., 2023).

### 5.2 Soft skills and potential covariate levels

No universal criteria exist to categorize resilience and empathy soft skills or potential covariate levels, and while specific cut-off points are available for certain Latin American programs (Díaz-Narváez et al., 2023; Díaz-Narváez et al., 2022; Díaz-Narváez et al., 2021; Reyes-Reyes et al., 2021), general standards for health sciences are lacking. Assuming that optimal professional development requires high levels of positive constructs and low negative affect, our results fall primarily within the average range, with ecological resilience at the lower limit of “high.” These findings are deemed unsatisfactory, indicating a developmental deficit in the constructs studied.

#### 5.2.1 Resilience

Results indicate that students possess limited capacities to resist, recover, and adapt to negative events or “turbulences” (Díaz-Narváez et al., 2025a,b). Ecological resilience allows for an initial resistance to stressors, yet this equilibrium is temporary and eventually leads to system instability or “erosion” if the disturbance persists. Once the system’s balance is compromised, engineering resilience operates as the mechanism for recovering the pre-existing equilibrium. However, persistent stressors—such as the COVID-19 pandemic (Sancho-Cantus et al., 2023)—require adaptive resilience. Rather than mere resignation, this represents an active psychological response aimed at managing and accommodating ongoing pressure without destabilizing the student’s psychological balance. Collectively, the data suggests these adaptive mechanisms are currently overextended or underdeveloped in the studied population.

#### 5.2.2 Empathy

Mean scores across all empathy dimensions indicate insufficient development (Díaz-Narváez et al., 2024; Huerta-González et al., 2024). Deficits in Compassionate Care (CC) could limit students’ capacity for spontaneous helpfulness by hindering their ability to share the patient’s emotional state. Similarly, low Perspective Adoption (PA) reflects a diminished capacity for the rational or intellectual understanding of a patient’s experiences and internal motives. Finally, the decrease in SIPS scores might reveal significant difficulty in connecting with the patient’s subjective thoughts and emotional needs (Díaz-Narváez et al., 2024). Consequently, students struggle to transcend their own perspectives to represent the patient’s cognitive and emotional reality accurately.

#### 5.2.3 Positive and negative affect

Positive (PA) and negative affect (NA) are independent dimensions of emotional experience (Ruiz-Pérez et al., 2020; Díaz-García et al., 2020). PA motivates adaptive approach behaviors (e.g., gratitude, hope), while NA reflects distress and unpleasant engagement (e.g., anxiety, sadness). Our results show a deficit in both dimensions, which may impair empathetic behavior and prosocial conduct. Effective regulation of positive emotions can typically buffer NA (Förster and Kanske, 2022); however, the observed imbalance—similar to patterns seen in social anxiety (Morrison et al., 2016)—suggests a specific disconnect between the students’ emotional state and their capacity for Compassionate Care (CC) within empathy.

#### 5.2.4 Family functioning

Family functioning, defined by cohesion and adaptability, is directly associated with both familial and individual resilience (Spitz and Steinhausen, 2023). While high levels of affective bonding and flexibility generally predict greater resilience, our findings show average scores. This suggests compromised emotional ties and rigid responses to turbulence. Interestingly, although extreme family styles have been linked to higher empathy in some contexts (Dávila Pontón et al., 2019), the overall mid-range values in this study point toward an underdeveloped socio-emotional foundation in the evaluated sample.

#### 5.2.5 Satisfaction with life

Evidence of a positive correlation exists between satisfaction with life and resilience (Shi et al., 2015) however, this relationship is bidirectional. Satisfaction with life serves as a critical foundation for resilient behavior. While the capacity to overcome adversity enhances well-being, evidence suggests that high levels of satisfaction with life provide students with the psychological resources (such as optimism and tranquility)—necessary to navigate high-pressure environments (Shi et al., 2015). According to the transactional model of stress, satisfied individuals are more likely to find positive meaning in adversity, effectively using their well-being to buffer the negative impact of external stressors. This suggests that satisfaction with life is not merely an outcome of success but a protective cognitive resource. For instance, when burnout (Duarte et al., 2022) threatens a student, an established baseline of satisfaction with life facilitates resilience by reinforcing the student’s ability to maintain normal functioning despite academic demands. The sample of first year students reported mean values of satisfaction with life of 25. Although improving these values might not fall within the direct responsibility of educational institutions, measuring these values could aid in the development of risk profiles and identify students that might be more and less susceptible to improving soft skills with academic training.

### 5.3 Soft skills and potential covariates

Soft skills have been the subject of extensive theoretical discussions. Various definitions and terms have been proposed (Espina-Romero et al., 2023; García et al., 2025), each encompassing “know-how-to-be” socio-emotional, and psychosocial skills. The vast majority of constructs associated with personality traits, values, emotional management, relationships, and the capacity to interact appropriately within one’s social environment overlap across different definitions (Gómez-Chaparro et al., 2024). The variables characterizing this term include self-awareness, resilience, self-regulation, autonomy, self-control, self-esteem, empathy, perseverance, and collaboration, according to some authors (Jardim et al., 2022; Kodali et al., 2025; Sylvia, 2025; van den Beuken et al., 2025).

However, complex overlaps (interactions) exist between these variables and others not previously listed. Indeed, some of these characteristics possess both cognitive and affective or emotional components, such as empathy (Hojat et al., 2001). Self-control cannot materialize without resilience (the capacity to resist, overcome, and adapt to negative events) (Mendez-Hurtado et al., 2025). Emotional self-regulation is one of the functions of the cognitive component of empathy and resilience, which also involves emotional regulation processes (Kong et al., 2018). The functions of emotional regulation reside in the brain’s orbitofrontal structure, which is common to both empathy and resilience. Therefore, “resilience has a significant positive effect on an individual’s empathic capacity, effectively reducing compassion fatigue and increasing empathic satisfaction” (Hegney et al., 2015; Ulloque et al., 2023). A deficit in this regulation would imply a lower capacity for empathy and resilience (Duarte et al., 2022; Espina-Romero et al., 2023), which could lead to a deficit in self-control and self-regulation (Nakhostin-Khayyat et al., 2024; Thompson et al., 2019). Consequently, when studying skills, competencies, and attitudes (among other human characteristics), it is complex to assert that studying such traits and their underlying variables in isolation can provide a complete answer to the situation examined. It only brings us closer to understanding, for example, soft skills.

Furthermore, it has been postulated that family functioning is also related to the development of empathy and resilience. Indeed, a family that exhibits balance across dimensions classified by cohesion is characterized by emotional bonding, support, and a sense of belonging. Families with flexibility are characterized by their adaptation to change, leadership, roles, and established rules (Dávila Pontón et al., 2019; Zicavo et al., 2012). When the characteristics associated with these two dimensions are negatively altered, the balance is disrupted, leading to family dysfunction (Gamarra Aparicio, 2025). This dysfunction may have exogenous or endogenous causes (or both) that can determine family attitudes and behaviors, affecting, for instance, the normal development of resilience and empathy (especially in the offspring) of one or more family members (Shi et al., 2015; Trong Dam et al., 2023; Wang et al., 2022; Zhang et al., 2024). However, the aforementioned relationships are complex, and observations of them may only be approximations of how family structure can relate to the attributes associated with the socio-emotional skills construct.

### 5.4 Educational implications

The results suggest that gender-responsive educational interventions could be essential for the balanced development of soft skills in health science students, especially when considering that sub-dimension of the soft skills might interact positively or negatively between each other (Díaz-Narváez et al., 2025a,b,c). Given that men scored higher in care with compassion and resilience adaptation (engineering), while women showed higher perspective-taking, educators should design curricula considering these specific gaps. For instance, incorporating perspective-taking exercises in male-dominated cohorts and fostering structural resilience in female-dominated environments could create a more holistic soft skill professional profile. Since the “engineering” dimension of resilience showed the most prominent effect size, this is important for intervention designers of Enhanced Stress Resilience Training, which should take sub-dimensions and gender differences into account to improve the effectiveness of the intervention (Sanders et al., 2025).

Furthermore, the significant correlations between resilience, empathy, and positive affect indicate that soft skills do not develop in a vacuum but function within a complex emotional system. In alignment with the broaden-and-build theory, positive emotions and satisfaction with life (*r* = 0.368) act as foundational resources that facilitate the acquisition of enduring personal competencies (Cohn et al., 2009). For educational institutions, this implies that monitoring these potential covariates is as critical as the soft skill training itself. By identifying students with low satisfaction with life or high negative affect early in their training, educators can provide targeted support to prevent the “soft skill decline” often observed in rigorous health programs. Risk profiling could aid identify and monitor students at risk of soft skill decline. Finally, accounting for these covariates in educational interventions will allow for a more accurate assessment of student progress and help mitigate confounding factors that might otherwise obscure the effectiveness of SEL programs (Sanders et al., 2025).

### 5.5 Limitations and strengths

Some limitations of the study must be acknowledged. Firstly, the sampling was considered a convenience sampling, which implies the potential for selection bias to affect the representativeness of the sample. In addition, the cross-sectional design implies our findings should be interpreted as correlations and not as causal relationships. Finally, data was obtained through a self-reported questionnaire, which might be susceptible to response bias. Despite these limitations, to the best of our knowledge, this is the first study looking at soft skills and potential covariate in first year health science students at the beginning of their training. Furthermore, we conducted robust psycho-metric evaluations of each scale structural validity, reliability with a sex invariance, a cut-off points analysis before evaluating sex differences and correlations.

## 6 Conclusion

The analysis indicates that the studied soft skills and their potential covariates function as a complex system. Consequently, examining empathy and resilience as core components of soft skills requires a multidimensional analytical approach. Within the framework of the broaden-and-build theory, which posits that positive emotions facilitate the accumulation of enduring personal resources, it is argued that cohesion, flexibility, positive affect, and satisfaction with life may enable health science students to acquire and sustain soft skills more effectively throughout their professional training (Sanders et al., 2025). From a neurobiological perspective, individual constructs such as affect, empathy, resilience, and satisfaction with life manifest as functional representations of a single integrated prefrontal-limbic-striatal-hippocampal axis. Family functioning acts as the external systemic context that relates to internal processes through interpersonal neural synchrony and neurochemical modulation, thus establishing the essential relational framework for the cultivation of interpersonal skills in the student.

These neurobiological considerations are not intended as explanatory findings of the present study, but rather as contextual elements derived from previous literature. Given the cross-sectional and self-report design, the present results should be interpreted primarily within a psychosocial and educational framework.

Our findings suggest that measuring the potential covariates could aid identify students that are more susceptible to learning and maintaining soft skills through their studies. Furthermore, it suggests that these covariates might be relevant to study the effects of interventions aimed at developing these soft skills as they might confound the relationship between the intervention and the soft skill developmental outcomes (Cohn et al., 2009). Future research should consider a longitudinal design that evaluates to what extent first year covariate values prevent soft skills decline or even predict higher change in soft skills during health sciences students training.

## Data Availability

The datasets presented in this study can be found in online repositories. The names of the repository/repositories and accession number(s) can be found at: OSF repository: https://osf.io/v5d3p/files/osfstorage?view_only=c457bac0642f431cb1d0ea11254e6cd3.
